# Relevance of Bile Acids in Cholangiocarcinoma Pathogenesis: Critical Revision and Future Directions

**DOI:** 10.3390/cells12121576

**Published:** 2023-06-07

**Authors:** Valentina Cossiga, Maria Guarino, Mario Capasso, Filomena Morisco

**Affiliations:** Diseases of the Liver and Biliary System Unit, Department of Clinical Medicine and Surgery, University of Naples “Federico II”, 80131 Naples, Italy; valentina.cossiga@gmail.com (V.C.); maria.guarino86@gmail.com (M.G.); mario.capa05@gmail.com (M.C.)

**Keywords:** cholangiocarcinoma, bile acid, liver carcinogenesis, gut microbiota, biliary microbiota

## Abstract

Cholangiocarcinoma (CCA), a highly heterogeneous cancer, is the second most common type of primary liver cancer. It is characterized by resistance to therapy and poor prognosis, with a 5-year survival rate lower than 20%. The pathogenesis of CCA is complex and multifactorial, and in recent years, bile acids (BAs) have been implicated in CCA development and prognosis. BAs belong to a category of amphipathic compounds that hold significant importance as signaling molecules and inflammatory agents. They possess the ability to activate transcriptional factors and cellular signaling pathways, thereby governing the regulation of lipid, glucose, and energy metabolism in diverse human disorders. These disorders encompass chronic liver diseases among other conditions. In this review, we provided an update on the current knowledge on the molecular mechanisms involving BAs in cholangiocarcinogenesis. Additionally, we analyzed the role of gut and biliary microbiota in CCA pathogenesis. Future research is required to better understand how to modulate BA activity and, possibly, identify new therapeutic strategies.

## 1. Introduction

Cholangiocarcinoma (CCA) is the second most common primary liver cancer after hepatocellular carcinoma (HCC), comprising approximately 15% of all primary hepatic malignancies and 3% of gastrointestinal tumors [1,2]. CCA includes a heterogeneous group of cancers emerging at any point belonging to the biliary tree and anatomically is classified as intrahepatic (iCCA), perihilar (pCCA) or distal (dCCA). pCCA and dCCA can also be collectively defined as “extrahepatic” (eCCA). pCCA is the most common subtype, accounting for 50–60% of all cases, followed by dCCA (20–30%) and iCCA (10–20%) [3]. CCAs typically manifest as asymptomatic during the early stages, leading to frequent diagnosis at advanced stages characterized by a worse prognosis. Although CCA is a rare tumor, its incidence (0.3–6 per 100,000 inhabitants per year) and mortality (1–6 per 100,000 inhabitants per year) have increased in the last years [1]. Moreover, despite advances in diagnosis and therapies, prognosis has not improved substantially in the past decade, with 5-year survival rates of 7–20% [4,5]. The pathogenesis of CCA is complex and multifactorial. Cholangiocarcinogenesis is coordinated by a complex interaction of extracellular ligands present in the tumor microenvironment (such as bile acids, growth factors, and pro-inflammatory cytokines) and augmented expression or defective activation of intracellular signaling pathways, causing cell proliferation and genetic/epigenetic modifications [2].

A causal relationship between bile acids (BAs) and tumors was firstly described in 1940 [6], but only in recent years, a role of BAs in cholangiocarcinogenesis has been elucidated. BAs play a central role as potential tumor-promoting actors and in controlling the proliferation of tumor cells of different origin [7]. The aim of this review is to furnish an overview of the principal mechanisms linking BAs to the onset and progression of CCA.

## 2. Bile Acid Physiology

### 2.1. Bile Acid Synthesis

BA synthesis mainly occurs in the liver through different pathways and several reactions, starting with the enzymatic conversion of cholesterol molecules. This phenomenon proceeds through two distinct pathways, namely the classical (or neutral) and the alternative (or acidic) routes, involving a minimum of 17 hepatic enzymes. The classic pathway is the major pathway and is responsible for about 90% of total BA production in the liver. The first biochemical reaction is catalyzed by 7α-hydroxylase (CYP7A1), converting cholesterol to 7α-hydroxycholesterol. This is a crucial enzyme because the BA synthesis rate is mainly controlled by transcriptional regulation of its gene [8]. The alternative pathway produces less than 10% of the total BAs in humans. In this pathway, the mitochondrial enzyme sterol 27-hydroxylase (CYP27A1) catalyzes the conversion of cholesterol into 27-hydroxycholesteol [9].

The primary BAs are cholic acid (CA) and chenodeoxycholic acid (CDCA). Both are mainly produced in the classic way, otherwise the alternative way only produces CDCA. Subsequently, the BAs are conjugated with either glycine (the major part) or taurine [10,11].

Once synthesized and conjugated, BAs are transported into bile, mainly by ATP-dependent cassette transporter (BSEP), and they are stored in gallbladder. Food intake stimulates the release of BAs into the intestine. Along the small intestinal tract, micellar BAs work as effective detergents to help the solubilization of fatty acids and monoacylglycerols, digestion, and the assimilation of dietary lipids and fat-soluble vitamins [9]. Subsequently, more than 90% of BAs are reabsorbed in the ileum and transported back to the liver through the portal system for re-secretion in the bile. This process is called entero-hepatic circulation of BAs [12,13].

In the small and mainly in the large intestine, a small percentage (about 5%) of BAs are deconjugated and dehydroxylated by bacteria via bile salt hydrolases, losing their ability to act as detergents. This process leads to the conversion of primary BAs into secondary BAs, more hydrophobic and toxic to humans. Specifically, CA and CDCA are converted to deoxycholic acid (DCA) and lithocholic acid (LCA). DCA undergoes passive reabsorption within the large intestine, while a significant portion of LCA is sulfated within the intestine and subsequently excreted in fecal matter. Furthermore, within the human intestine, LCA has the potential to undergo conversion into more soluble and less toxic compounds, such as hyocholic acid (HCA) and ursodeoxycholic acid (UDCA) [14,15,16].

### 2.2. Physiological Bile Acid Functions

BAs, together with cholesterol and phosphatidylcholine, form the lipid phase of bile. They are amphipathic detergent molecules formed by a polar group and an apolar part. The conjugation process makes them negatively charged molecules with increased solubility, and when their concentration increases, conjugated BAs form micelles able to solubilize cholesterol. This function concurs with cholesterol homeostasis and favors fat and liposoluble vitamin absorption in the intestine. They also sustain canalicular bile flow through specific BA export pumps, regulating the extrusion of BAs linked with water in biliary canaliculi, determining the “BA-dependent bile flow” and facilitating biliary secretion of xenobiotics and endogenous metabolites [15,17].

In addition to their digestive role, BAs act as signaling molecules and as ligands of many receptors that can modulate fundamental metabolic and cellular function. BAs directly activate nuclear receptors (NR), such as pregnane X receptor (PXR), farnesoid X receptor (FXR), and vitamin D receptor (VDR), which act by limiting BA synthesis [14]. FXR, mainly expressed in the liver, can be activated by LCA, DCA, and CDCA. FXR activation leads to negative feedback, suppressing BA synthesis by inducing other NRs, which inhibit hepatic CYP7A1 gene expression. This interaction also inhibits ileal BA reabsorption. Moreover, FXR induces the intestinal transcription of fibroblast growth factor 15 (FGF15), responsible for BA synthesis suppression. In this scenario, FXR plays a role in the control of BA synthesis and transport [18,19]. PXR can be activated by secondary BAs and by a wide range of xenobiotics (such as rifampicin) because of the hydrophobic ligand pocket. The pathway, caused by PXR activation, represses hepatic CYP7A1 expression and induces intestinal FGF15, suggesting a role similar to FXR, with differences in the ligand involved [20]. BAs are also potent endogenous ligands of VDR, whose activation promotes the inhibition of CYP7A1 transcription and induces ATP-binding cassette transporters, all favoring the excretion of BAs [21].

Another metabolic role of BAs is their contribution to the control of glucose and lipid homeostasis. One method of metabolism regulation is signaling through G protein-coupled receptors (GPCRs), in particular Takeda G protein-coupled bile acid receptor (TGR5) and sphingosine-1-phosphate 2 receptor (S1PR2). TGR5 can be activated not only by conjugated and unconjugated primary BAs but also by secondary BAs, such as LCA, which is one of the most potent activators of this receptor. Ligand binding to TGR5 triggers activation leading to an increase in intracellular adenosine monophosphate (cAMP) concentration to modulate glucose homeostasis through glucagon-like peptide 1 (GLP1) secretion and to promote energy metabolism; furthermore, TGR5 couples with stimulatory G proteins (Gs) to modulate cell proliferation. In the literature, substantial evidence has been presented regarding the significant role of TGR5 in supporting the proliferation and growth of bile duct epithelial cells in a bile acid (BA)-dependent manner [22,23]. Conjugated BAs can also activate S1PR2, inducing the activation of protein kinase B (AKT) and extracellular signal-regulated kinase 1–2 (ERK1/2) cellular signaling pathways, with the subsequent increase in nuclear sphingosine-1-phosphate (S1P). S1P is a lipid mediator that regulates various cellular processes, such as cell proliferation, angiogenesis, endothelial barrier integrity, and inflammation. Nuclear S1P inhibits specific histone deacetylases (HDACs) determining an upregulation of genes encoding NRs and enzymes involved in glucose and lipid metabolism. This pathway is also involved in the downregulation of gluconeogenesis genes as insulin-like activity in the liver [24,25].

**Figure 1:**
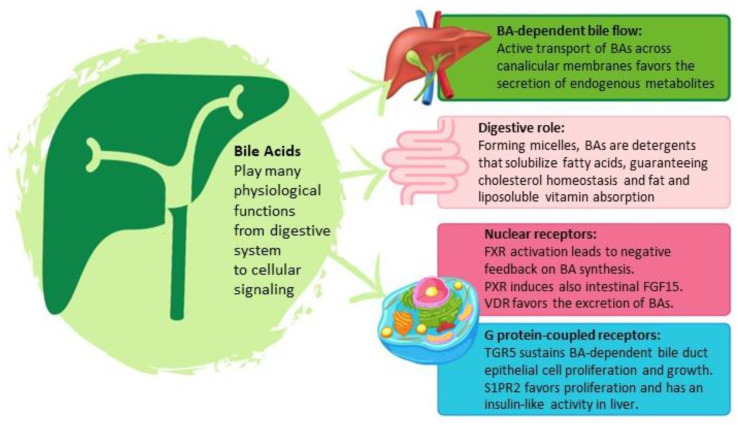
Physiological BA functions. BAs (bile acids), FXR (farnesoid X receptor), PXR (pregnane X receptor), VDR (vitamin D receptor), TGR5 (Takeda G protein-coupled bile acid receptor), S1PR2 (sphingosine-1-phosphate 2 receptor).

In summary, BAs have many key roles in humans (Figure 1). Their synthesis and turn-over favor cholesterol homeostasis, and their emulsifying capacity allows for the absorption of nutrients into the intestine. BAs also play a crucial role as a signaling molecules, regulating different cellular processes involved in cell growth and metabolism. 

## 3. Bile Acids Act like a Double-Edged Sword in Cholangiocarcinogenesis

The pathogenesis of CCA is a multistep process involving oxidative stress and inflammation, mutagenic processes with genetic and epigenetic factors, as well as environmental proliferative stimuli (i.e., lifestyle, diet, exposure to environmental toxics) with an intricate network of oncogenic mechanisms, creating heterogeneous tumors [2]. Cholestasis has been identified as one of the major risk factors for the development of CCA. In this context, recent advances have unveiled BAs’ multifaceted involvement beyond their fundamental role in bile formation and secretion. These molecules have been shown to be able to protect against CCA or promote its development. The intrahepatic accumulation of BAs does not seem to have a direct carcinogenic effect but may favor CCA development toward a cocarcinogenic role, inducing cholangiocyte inflammation and bile duct proliferation as well as enhanced inflammation and reduction in FXR-dependent chemoprotection [26]. Accordingly, several studies showed that an imbalance of lipids and “toxic BAs” in bile may have a pathogenic role in carcinogenesis [27,28,29]. As a consequence, serum BA composition could be used as a biomarker of CCA since the differences between patients with and without CCA seem to be due to an altered primary bile acid biosynthesis and a different bile secretion pathway [30]. Farhat et al. showed that higher serum concentrations of conjugated BAs and low levels of unconjugated BAs were associated with an increased risk of CCA and lethal liver disease, suggesting that the accumulation of BAs could be involved in cancer development and liver disease progression [31,32].

The imbalance of lipids and “toxic BAs” in bile seems to be created by the activation of peroxisome proliferator-activated receptor-a (PPARa). As a matter of fact, PPARa agonists, used for treating metabolic syndrome, are associated, in animal studies, with increased synthesis of BAs, hepatic damage, cholangiocyte proliferation, and liver carcinogenesis [33].

**Figure 2:**
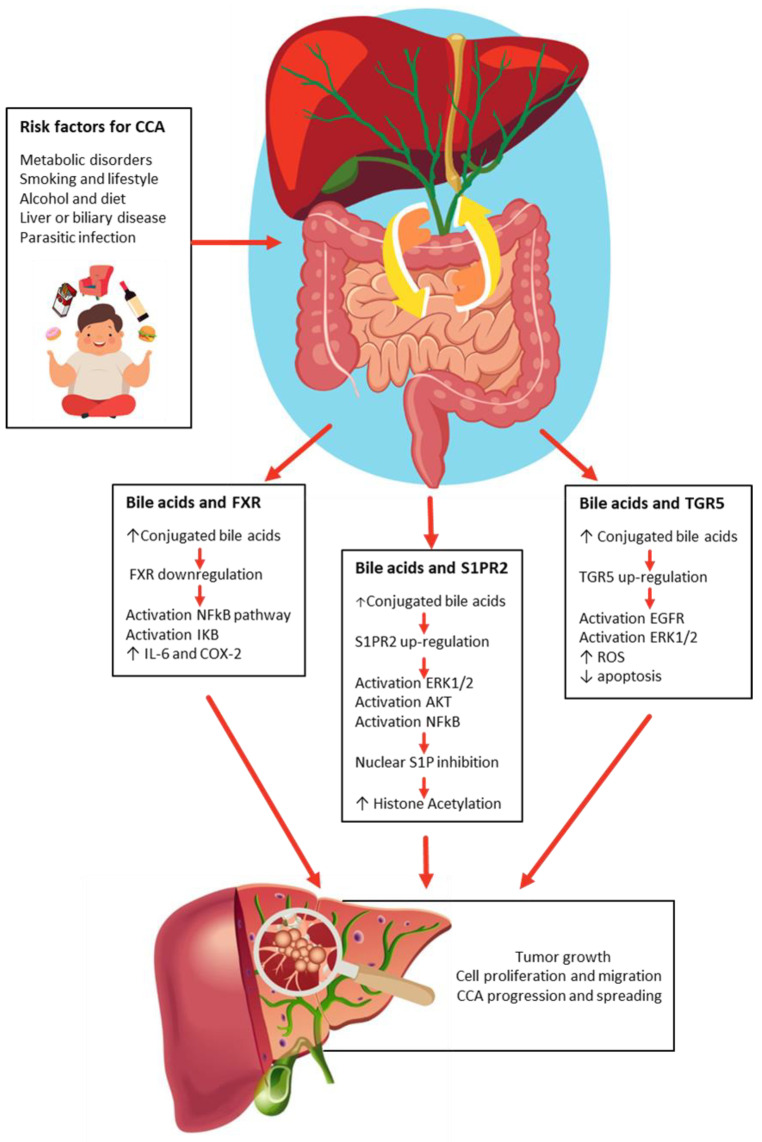
The BAs-FXR-S1PR2-TGR5 axis.

BAs have both hydrophilic and hydrophobic surfaces, with a protective and less cytotoxic role played by the hydrophilic surface, while the hydrophobic part of BAs causes cell damage, inducing oxidative stress and genomic instability, modulating the expression of tumor-suppressor/-promoting genes. BAs exhibit an additional role as signaling molecules, effectively inducing the proliferation of liver cancer cells. BAs engage in binding interactions with both nuclear and membrane receptors, with varying affinities, notably involving FXR, S1PR2, and TGR5 (Figure 2) [34,35,36].

### 3.1. FXR and Bile Acids

Farnesoid X receptor (FXR) is a nuclear hormone receptor for BAs, which is so important in their homeostasis that it is considered the intracellular sensor of BAs. The FXR expression is elevated in cells exposed to high BA concentrations, such as cholangiocytes and enterocytes, and above all, FXR maintains BA concentrations within a physiological range to prevent their accumulation and the associated cellular damage [37]. Moreover, there are four FXR isoforms, whose differential activation depends on the BAs’ pool composition as well as their expression pattern [38]. Consequently, within the liver, the FXR receptor plays a pivotal role as a protective factor against CCA by virtue of its involvement in preserving BA homeostasis. This protective role stems from its ability to restore BA balance following liver injuries, promote hepatocyte protection, enhance cell survival, have anti-inflammatory properties, and modulate gene expression. Notably, FXR contributes to the upregulation of tumor-suppressor genes while inhibiting the transcription of oncogenes [39,40]. Supporting its protective role, a recent study by Erice et al. demonstrated that the expression of FXR is significantly reduced in CCA tissue compared to nontumor tissue, and this downregulation correlates with the degree of tumor cell differentiation. Moreover, their study revealed that the FXR agonist, obeticholic acid (OCA), effectively inhibits CCA tumor growth in immunodeficient mice. In vitro experiments further revealed that CCA human cells exhibit downregulated FXR expression, and OCA treatment leads to the inhibition of CCA cell proliferation, migration, and mitochondrial energy metabolism. Thus, reduced FXR expression alone is insufficient to promote the onset of CCA or sustain cancer cell proliferation unless accompanied by elevated levels of BAs. While FXR deficiency appears to play a role in promoting cancer, an increase in BA levels is required for the stimulation of cell proliferation and the formation of cancer [41].

In in vitro studies, FXR activation could strongly suppress the activity of the nuclear factor-kappa B (NF-kB) [42]. Accordingly, it has been showed that free BAs inhibited the cell proliferation and conjugated BAs stimulated cell proliferation, activating or blocking the NF-kB pathway, respectively [43]. In particular, Dai et al. showed in mice models of CCA that conjugated BAs stimulated tumor growth, upregulated the phosphorylated kinase inhibitor B (IkB), and increased interleukin-6 (IL-6) and cyclooxygenase-2 (COX-2), while free BAs decreased tumor growth, downregulated the phosphorylated IkB, and decreased IL-6 and COX-2 [44]. Because free unconjugated BAs have been shown to upregulate FXR in CCA cells, it is possible that FXR expression is dysregulated as a result of the inability of CBAs to enter in CCA cells to activate this nuclear receptor [35].

Moreover, Sirtuin 1, another regulator of BA homeostasis, appears to play a crucial role in modulating the regenerative response of the liver through post-transcriptional modifications, including acetylation of FXR and neighboring histones, which potentially contribute to CCA onset by disrupting the homeostasis of BAs via persistent FXE deacetylation. This suggests that the dysregulation of BA homeostasis and subsequent CCA development may be influenced by the aberrant deacetylation of FXR facilitated by Sirtuin 1 [45]. Similarly, the tumor-suppressive role of microRNA-22 (miR-22) is regulated by FXR expression in the liver: chenodeoxycholic acid, due to its high affinity for FXR, increases miR-22 levels in liver cells with a silencing effect on cyclin A2 [46].

Finally, alcohol is able to downregulate FXR, with a subsequent increase in BA synthesis and the liver BA pool. So, chronic alcohol intake is also able to deeply affect the entero-hepatic circulation of BAs through effects on BA transporters, leading to increased serum levels of BAs [47,48].

### 3.2. SIPR2 and Bile Acids

The sphingosine 1-phosphate (S1P) pathway is involved in the inhibition of the apoptosis of biliary cancer cells [49] and in CCA progression. In a rat model of CCA that closely mimics the human disease, Dumur CI et al. revealed a noteworthy correlation between the progressive increase in tumor sphingosine kinase 1 (SphK1) expression and enhanced tumor growth, along with the development of malignant bile duct obstruction [50]. These effects are obtained by BA stimulation of the S1P receptor 2 (S1PR2), the predominant S1PR isoform in hepatocytes, cholangiocytes, and CCA cells [36]. S1P elicits its biological function either as an intracellular signaling molecule or as an agonist of S1PR2 [51]. The stimulation of S1PR2 seems to play a crucial role in conjugated BA-mediated invasive growth, bile duct proliferation, and upregulation of COX-2 expression and prostaglandin E2 production in CCA cells [17,52,53]. Accordingly, in the study conducted by Liu et al., it was demonstrated that both the mRNA and protein levels of S1PR2 are markedly elevated in human CCA cells. Additionally, the expression of S1PR2 was found to be significantly higher in CCA tissue compared to nontumor tissue. Moreover, the activation of the ERK1/2 and AKT signaling pathways was observed in CCA cells upon exposure to conjugated BAs. Taken together, all these results strongly suggest that S1PR2 is a key regulator of CCA cell growth stimulation and cell migration/invasion caused by conjugated BAs [35]. Furthermore, in the study conducted by Wang et al., it was revealed that the expression of S1PR2 is dose-dependently increased in response to BAs. The elevated levels of BAs result in the activation of S1PR2, subsequently triggering the activation of the ERK1/2 signaling pathway. This activation leads to the phosphorylation of nuclear sphingosine kinase 2 (nSphK2), which in turn controls the levels of nuclear S1P. Nuclear S1P acts as a potent inhibitor of histone deacetylases (HDACs), promoting histone acetylation and thereby enhancing the transcriptional activity of key genes involved in cell growth regulation and hepatic lipid metabolism. Moreover, the activation of ERK1/2 can also induce the activation of NF-kB, a transcription factor that promotes the expression of various inflammatory genes, including COX-2 [54].

### 3.3. TGR5 and Bile Acids

TGR5 (G-protein-coupled BA receptor 1) is a membrane-bound receptor responsive to BAs, expressed in several non-parenchymal cells of the liver, including sinusoidal endothelial cells, Kupffer cells, gallbladder epithelial cells, and cholangiocytes, even if its expression level is much lower than that of S1PR2 [54]. TGR5 protects from BA toxicity by preventing death-receptor-mediated apoptosis [55]. Moreover, cholangiocyte proliferation is enhanced by BAs, which are potent TGR5 ligands. BA-dependent TGR5 activation can trigger cholangiocyte proliferation as well as cell protective and antiapoptotic effects, which may delineate important mechanisms to protect cholangiocytes from higher BA concentrations, modulating the bile composition and stimulating the generation of the so-called “bicarbonate biliary umbrella”, which seems crucial to avoid BA-induced toxicity [56]. While TGR5 acts with cytoprotective effects in nontumoral cholangiocytes, in malignant cells, the same receptor may confer apoptosis resistance and enhance proliferation through an increase in reactive oxygen species (ROS) and may thus modulate cancer progression, since it has been linked to cholangiocyte proliferation induced by epidermal growth factor receptor (EGFR) and ERK1/2. Additionally, the antiapoptotic effects determined by the activation of TGR5 are mediated by serine phosphorylation of the CD95 death receptor in CCA cell lines and murine cholangiocytes [23]. So, if FXR is a tumor suppressor, TGR5 favors carcinogenesis by stimulating cell proliferation and survival. Indeed, Erice et al. showed that TGR5 expression is upregulated in CCA tissue compared to nontumor tissue, correlates with perineural invasion, and stimulates the proliferation, migration, and mitochondrial energy metabolism of CCA cells in vitro [36]. Confirming these data, Song et al. showed that the gene expression levels of TGR5 and S1PR2 were greatly enhanced in CCA cells treated with conjugated BAs [53].

In conclusion, the BAs-FXR-S1PR2-TGR5 axis needs to be considered within the overall framework of cholangiocarcinogenesis, taking into account that BAs are involved in the regulation of apoptosis resistance, proliferation, and preservation of ductal bile secretion during damage (Figure 2). Conversely, during CCA, the proliferative and antiapoptotic effects of BAs on biliary epithelium may result in harmful effects. The mechanisms implicated in protection and damage by BAs on the biliary tract need to be elucidated.

## 4. The Mutual Interaction between BA and Microbiota

### 4.1. Gut Microbiota

The human gut is colonized by a complex and dynamic range of symbiotic microorganisms, collectively termed the “gut microbiome”, which is one of the most complex and densely populated ecosystems known. The gut microbiota has the role of defending the human host from pathogens and maintaining immune balance and metabolic homeostasis [57]. The great majority of bacteria colonizing the human gut are obligate anaerobes, and in particular, species in the *Firmicutes* and *Bacteroidetes* makes up more than 90% of the human gut microbiota [58]. Even though the gut microbiome promptly benefits the host, it can be also involved in the development of diseases, and growing evidence is emerging about its involvement in both local and distant carcinogenesis both in humans and animal models [59,60].

**Figure 3:**
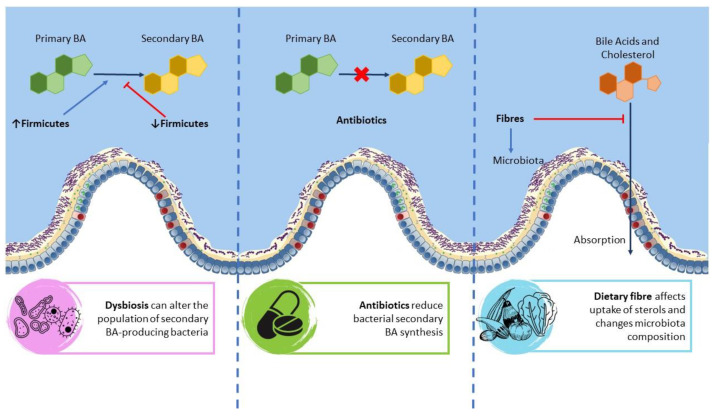
The mutual interaction between BAs and gut microbiota.

Due to its close connection to the gut via the hepatic portal vein, the liver is the first recipient of gut microbiome molecules and metabolites, including BAs, choline, indole derivatives, lipopolysaccharide (LPS), and short-chain fatty acids (SCFAs) [61]. In particular, BAs seem to be a major regulator of gut microbiota [62]. In physiological conditions, the gut microbiota mediates BA metabolism by transforming primary BAs, synthesized by hepatocytes, into secondary and unconjugated forms [63]. The first step of secondary BA metabolism is hydrolysis via bile salt hydrolases (BSHs). BSHs are highly conserved across all major gut microbiota phyla but are different between bacteria because of their preferential activity towards either glycine-conjugated or taurine-conjugated BAs [64]. The mutual interaction between BAs and gut microbiota seems to be central in many diseases. In fact, the level of BA entering the large intestine thus has a deep effect on the major division-/phyla-level taxa in the gut lumen. For example, decreased levels of BAs in the gut favor gram-negative members of the microbiome, some of which produce LPS, while increased BA levels in the gut favor gram-positive members of the *Firmicutes*, including bacteria that dehydroxylate primary BAs into secondary toxic BAs [65]. On the other side, changes in gut microbiota composition, induced by diet, drugs, age, and diseases, influence the BA pool due to their effect on the bile-salt-metabolizing members of the microbiome (Figure 3) [66].

A direct relationship between the gut microbiota, secondary BAs, and liver cancer has been suggested by recent studies, and two pathways involved in the gut microbiota regulation of liver cancer via secondary BAs have been described [67]. Ma C. et al. showed, in mice, that primary BAs modulate the survival of natural killer T (NKT) cells in the liver, inducing interferon-gamma production and tumor growth inhibition. On the contrary, secondary BAs, produced mostly by *Clostridium species*, reduce the accumulation of NKT cells, with subsequent tumor growth induction [63]. Moreover, Loo TM et al. demonstrated that changes in *Clostridium species* induced by various factors cause an increased production of secondary BAs. This increases cyclooxygenase-2 (COX-2) activity in hepatic stellate cells and prostaglandine-mediated suppression of antitumor immunity [68]. However, the exact mechanisms through which the gut microbiota mediates BA composition in CCA are still not clear.

Instead, several studies have investigated the gut microbiota composition in patients with CCA. Ito Z. et al. [69] evaluated fecal microbiota composition in 30 patients with biliary tract cancers (BTCs), in 11 patients with benign biliary diseases (BBDs), and in 10 age- and sex-matched healthy controls. In the BTC group, they found a higher presence of *Gammaproteobacteria*, such as *Enterobacteriaceae*, and a lower presence of *Clostridia* compared to the other groups. Some butyrate-producing bacteria were, instead, more abundant in healthy subjects. These different compositions of fecal microbiota suggest that the development of BTC can lead to changes in microbiota at different levels.

Jia X. et al. [70] analyzed gut microbiota composition in patients with iCCA, patients with hepatocellular carcinoma (HCC), patients with liver cirrhosis (LC), and in healthy controls. They found that the phylum *Firmicutes* was the most abundant in all the groups. Regarding the alpha- and beta-diversity, patients with iCCA had the highest biodiversity among all four groups. At genus level, patients with iCCA showed higher prevalence rates of *Lactobacillus*, *Actinomyces*, *Peptostreptococcaceae*, and *Alloscardovia* than the other groups. These results showed that gut microbiota composition is more different in CCA compared to HCC and healthy controls, probably due to the strict connection between the gut and cholangiocytes. Moreover, the authors analyzed the plasma–stool ratio of BAs and showed that the glycoursodeoxycholic acid and tauroursodeoxycholic acid plasma–stool ratios were increased in patients with ICC and were positively correlated with the genera *Lactobacillus* and *Alloscardovia*. Furthermore, patients with iCCA and vascular invasion have different gut composition compared to patients without vascular invasion, suggesting that the microbiota could be related to vascular invasion.

In brief, gut microbiota composition is significantly different in patients with CCA compared to patients with other liver diseases or controls. BAs are among the main factors responsible for this difference and could be involved in liver carcinogenesis through gut microbiota modification.

### 4.2. Biliary Microbiota

Bile has traditionally been considered sterile in subjects without prior biliary intervention or biliary disease. The chemical and physical properties of bile and its antimicrobial capacity were able to create a hostile environment for bacteria. Furthermore, the challenge of collecting bile samples joined with the lack of awareness of culture techniques in detecting microbes in low-charge samples supported this hypothesis for a long time [71,72]. Instead, recent studies reported that a microbial ecosystem does exist in patients with and without biliary diseases (Table 1) [69,73,74,75,76,77,78,79]. For example, Ito Z. et al. [69] analyzed biliary microbiota in a small subgroup of patients with BTC and BBD. They found that the percentage of bacterial species in bile microbiota differs among subjects, but the bile of patients with BTC was enriched in *Enterobacteriaceae* compared to patients with BBD. Moreover, they observed that bile samples had significantly lower sequencing depths than stool samples, reflecting their lower bacterial content. Another important study conducted by Miyabe K. et al. [76] analyzed stool and bile microbiota in patients with primary sclerosing cholangitis (PSC), CCA, and controls. They showed that bile and stool samples had different microbiota profiles but that the microbiota of the bile and stool samples from the same individuals were correlated. Moreover, CCA bile samples had a significant difference in species richness from control bile samples. In particular, CCA bile was significantly rich in *Firmicutes*, *Fusobacteria*, and *Actinobacteria* in comparison to control bile. It is remarkable that both *Fusobacteria* and *Firmicutes* were also increased in PSC patients in relation to the duration of the disease. Similar results on bile microbiota composition were obtained by Okuda S. et al. [77]. Moreover, they found that *Akkermansia* was detected in the majority of bile samples of patients with CCA but not in patients with pancreatic cancer, and its presence was related to a better outcome after biliary drainage.

However, the results of these studies are contrasting and limited by several factors. First of all, the standardization of the sampling methods should be evaluated. Different methods have been applied to perform bile sampling; with a high risk of contamination, sampling standards are currently lacking. Considering that studies on healthy human biliary microbiota are not feasible for ethical issues, comparative studies on the biliary microbiota of subjects with different biliary diseases could be able to identify the microbial fingerprint of each illness. Furthermore, samples are obtained from patients with CCAs in different stages, and comparison between the bile microbiota of these patients is not possible.

## 5. Conclusions

In conclusion, CCA pathogenesis is complex and multifactorial. This review provides a comprehensive overview of the role of BAs in liver carcinogenesis. These molecules not only play a pivotal role in bile formation and secretion but are also crucial in protective and harmful processes involving the biliary tract. In particular, in a CCA setting, BAs seem to promote cholangiocarcinogenesis through several mechanisms, such as the activation of proliferative pathways, the inhibition of apoptosis, the promotion of cell proliferation, and the modulation of gut and biliary microbiota. Currently, the pathways involved still need to be completely elucidated. In the future, the modulation of these mechanisms may warrant a better comprehension of genetic, acute, and chronic cholestatic human liver diseases and possibly suggest therapeutic strategies for these conditions.

## Figures and Tables

**Table 1:** Studies evaluating bile microbiota composition in healthy and CCA patients.

Author, Year	Biological Specimens	Sampling Methods	Conclusions
Healthy			
Molinero N, 2019 [73]	Bile and gallbladder tissue	Sterile sampling during liver transplants from liver donors	Bile samples and gallbladder tissues of individuals without gallstones are rich in *Actinobacteria*, *Firmicutes*, and *Bacteroidetes* as well as abundant in the *Propionibacteriaceae* family.
Cancer			
Saab M, 2019 [75]	Bile	ERCP	At phyla level, *Firmicutes*, *Fusobacteria*, and *Cianobacteria* levels were significantly lower in CCA than in the controls.
Ito Z, 2022 [69]	Bile and feces	ERCP or surgery	Bile of patients with cancer was enriched in *Enterobacteriaceae* compared to that in patients with benign biliary disease.
Miyabe K, 2022 [76]	Bile and feces	ERCP or surgery	The microbiota of the bile and stool samples from the same subjects were correlated. CCA bile was characterized by increased abundance of *Firmicutes*, *Fusobacteria*, and *Actinobacteria* compared to control bile samples. *Fusobacteria* and *Firmicutes* were also increased with increasing PSC duration in bile from patients with PSC.
Okuda S, 2022 [77]	Saliva, gastric and pancreatic juice, bile, feces, tumor and nontumor tissue	Surgery	*Akkermansia* was the most abundant, and it was present only in bile samples. *Akkermansia* was detected in 7/11 patients with biliary tract cancer and in none of the 4 patients with pancreatic cancer.
Aviles-Jimenez F, 2016 [78]	Epithelial cells from the biliary duct	Brushing during ERCP	In extrahepatic cholangiocarcinoma, the microbiota showed significant changes in microbial composition, with a predominant presence of *Phylum Proteobacteria* in all samples. *Fusobacterium*, *Prevotella*, *Actinomyces*, *Novosphingobium*, and *H. pylori* increased in patients with CCAs.
Zhou D, 2013 [79]	Serum and bile	ERCP	The infection rate of *Helicobacter* spp was significantly higher in CCA compared to in the control and benign biliary pathology groups.
Di Carlo P, 2019 [80]	Bile	ERCP	*E. coli*, *Pseudomonas aeruginosa*, and *Klebisiella pneumonas* were identified in the cancer and were associated with reduced survival time.

## Data Availability

Not applicable.
